# Habitat stability and occurrences of malaria vector larvae in western Kenya highlands

**DOI:** 10.1186/1475-2875-8-234

**Published:** 2009-10-21

**Authors:** Yousif E Himeidan, Guofa Zhou, Laith Yakob, Yaw Afrane, Stephen Munga, Harrysone Atieli, El-Amin El-Rayah, Andrew K Githeko, Guiyun Yan

**Affiliations:** 1 Centre for Global Health Research, Kenya Medical Research Institute (KEMRI), P.O. Box 1578, Kisumu 40100, Kenya; 2 Program in Public Health, University of California, Irvine, CA 92697, USA; 3 Department of Zoology, University of Khartoum, P.O. Box 321, Khartoum 1115, Sudan; 4 Faculty of Agriculture and Natural Resources, University of Kassala, New Halfa, Sudan

## Abstract

**Background:**

Although the occurrence of malaria vector larvae in the valleys of western Kenya highlands is well documented, knowledge of larval habitats in the uphill sites is lacking. Given that most inhabitants of the highlands actually dwell in the uphill regions, it is important to develop understanding of mosquito breeding habitat stability in these sites in order to determine their potential for larval control.

**Methods:**

A total of 128 potential larval habitats were identified in hilltops and along the seasonal streams in the Sigalagala area of Kakamega district, western Kenya. Water availability in the habitats was followed up daily from August 3, 2006 to February 23, 2007. A habitat is defined as stable when it remains aquatic continuously for at least 12 d. Mosquito larvae were observed weekly. Frequencies of aquatic, stable and larvae positive habitats were compared between the hilltop and seasonal stream area using χ^2^-test. Factors affecting the presence/absence of *Anopheles gambiae *larvae in the highlands were determined using multiple logistic regression analysis.

**Results:**

Topography significantly affected habitat availability and stability. The occurrence of aquatic habitats in the hilltop was more sporadic than in the stream area. The percentage of habitat occurrences that were classified as stable during the rainy season is 48.76% and 80.79% respectively for the hilltop and stream area. Corresponding frequencies of larvae positive habitats were 0% in the hilltop and 5.91% in the stream area. After the rainy season, only 23.42% of habitat occurrences were stable and 0.01% larvae positive habitats were found in the hilltops, whereas 89.75% of occurrences remained stable in the stream area resulting in a frequency of 12.21% larvae positive habitats. The logistic regression analysis confirmed the association between habitat stability and larval occurrence and indicated that habitat surface area was negatively affecting the occurrence of *An. gambiae *larvae. While *An. gambiae *and *An. funestus *larvae occurred throughout the study period along the streams, a total of only 15 *An. gambiae *larvae were counted in the hilltops, and no *An. funestus *were found. Moreover, no larvae managed to develop into adults in the hilltops, and the density of adult *An. gambiae *was consistently low, averaging at 0.06 females per house per survey.

**Conclusion:**

The occurrence of malaria vector larvae in the hilltop area was uncommon as a result of the low availability and high instability of habitats. To optimize the cost-effectiveness of malaria interventions in the western Kenya highlands, larval control should be focused primarily along the streams, as these are likely the only productive habitats at high altitude.

## Background

Anopheline larvae occupy habitats that vary widely in their physicochemical properties, surface areas and vegetation. Habitats can be highly species specific and extreme variation in larval production has been shown to occur on a small scale both spatially and temporally [1-5]. Informed larval interventions that target the more prolific habitats have been suggested to have great potential in combating malaria [6-9], especially at a local, rather than nationwide, scale. Intervention therefore needs to be adaptable to, and specific for local communities.

A recent series of malaria epidemics have occurred in the western Kenya highlands [10-12]. Although year-round the valleys constitute the majority of vector breeding sites, potentially important larval habitats can also be found in the uphill areas during the rainy season [13,14]. *Anopheles gambiae *is considered a typical pioneer species and it has been shown to be capable of colonizing these uphill habitats within just a few days of the rain falls [15]. This colonization by blood-fed female mosquitoes brings with it the risk of malaria infection to the immunologically naïve, uphill human population [16]. However, extending breeding site interventions to account for this spatial expansion in the vector population is not necessarily justified. Larvae of *An. gambiae *usually occur in small, temporary and sunlit pools [17-19], which are prone to drying up quickly [20]. In order to justify larval control in the uphill regions during the rainy season, it must be determined whether the transient pools of water in these locations are found are sufficiently stable for the locally recorded threshold of 12 days necessary for larvae to develop into adults [4]. It was the objective of this study to develop the knowledge of the association between habitat stability and vector productivity in the uphill regions of western Kenyan highlands, and to elucidate the role of these habitats in local malaria transmission.

## Materials and methods

### Study sites

**Figure 1:**
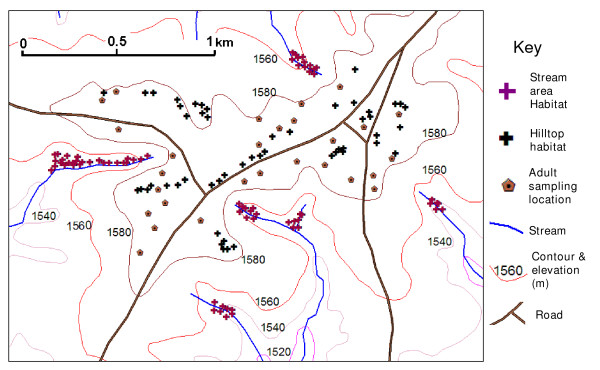
**Map showing the topography of the study area and locations of larvae and adult mosquitoes sampling**.

**Figure 2:**
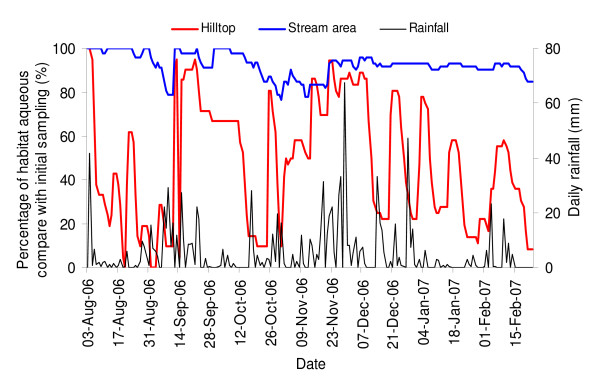
**Temporal variations in daily accumulative rainfall (mm) and the percentage of habitats in the stream areas and hilltops that were aqueous compared to the initial sampling period**.

The study was conducted in Sigalagala (0°12'N, 34°46'E) area in Kakamega district, western Kenya highlands with elevation of 1500-1600 m above sea level. Distinct topographical features of the study villages typically consist of hills and valleys. The hillside is mostly dotted with maize plantations. A few patches of indigenous forests are located along the valleys. Six different seasonal streams flow within the valleys in the study villages and empty into the main Yala river (Figure 1). The inhabitants live in traditional stick and mud houses with thatch or iron sheet roof. Most residences in the area are located uphill, with a few on the hillsides. The areas within 50 m of the nearest stream were considered to be the 'stream area', otherwise they were considered as 'hilltop' (Figure 1). The area experienced a short rainy season from August to November 2006 and the total rainfall was 1050.2 mm during the period of study from August 3, 2006 - February 23, 2007 (Figure 2).

### Habitat monitoring and larval sampling

A total number of 128 aquatic habitats were identified within the study area, with 47 in the hilltops and 81 in the stream area (Figure 1). These habitats were used as fixed observation sites throughout the study period and no additional habitats were added into the study after the initial stage. The habitats were mapped using a Global Positioning System (GPS) unit. Individual habitats were numbered and their surface area recorded along with the land use type. The water availability in each habitat was observed daily. Mosquito larval surveys were done weekly in all study sites. The immature stages were sampled using the standard dipping method (dipper size of 350 ml) [21]. Water was dipped up to 20 times. When a habitat was too small to make 20 dips, water was dipped as many times as possible. Mosquito larval species were identified [22], counted and returned to the habitats. Samples of young larvae of *An. gambiae s.l*. were identified to species using the polymerase chain reaction (PCR) [23].

### Adult sampling

Adult malaria vectors were monitored in the uphill area by collection from a randomly selected sample of twenty-eight houses (Figure 1). These 28 houses were fixed for adult samplings throughout the study period. All selected houses were numbered and geo-referenced using the GPS. Adult mosquitoes were collected biweekly from August 2006 to February 2007 using the indoor pyrethrum spray catches [22]. The mosquitoes collected were identified to species morphologically [17].

### Data analysis

The number of days that a habitat remained aquatic and the number of times that immature stages of *An. gambiae *were present for each individual habitat over the 205 d study period were recorded. To determine the impact of rainfall on habitat stability and larval occurrence, the study period was divided into two: the rain season lasting from August to the end of November, 2006, and the post-rain season lasting from the 1^st ^of December, 2006 to the 23^rd ^February, 2007. A habitat was defined as stable if it remained aqueous for 12 d or longer, following on from a previous study in the same area that showed the egg-to-adult development cycle of *An. gambiae *requires at least this length of time [4]. The mean densities of immature stages (larvae/dip) were calculated per habitat. The χ^2^-test was used to compare the frequencies of aquatic, stable and larvae positive habitats between hilltop and stream areas. Tukey-Kramer HSD test of repeated measure MANOVA was used to compare the mean occurrence of anopheline larvae between different seasons and different topographic areas. Multiple logistic regression with backward stepwise analysis was then performed to identify the factors which significantly influenced the occurrences (presence/absence) of *An. gambiae *larvae. Any independent variable with a significance level of < 95% was eliminated from the final model. The analysis was conducted using JMP statistical software (JMP SAS Institute Inc. 2003).

## Results

### Species composition and habitat types of malaria vector larvae

A total of 12,410 mosquito larvae were collected, of which 1,675 (13.5%) were *Anopheles spp*. Among the anopheline larvae collected, 61.3% (n = 1027) were *An. gambiae s.l*., 37.2% (n = 623) were *Anopheles funestus*, 0.8% (n = 14) were *Anopheles coustani*, 0.4% (n = 7) were *Anopheles squamosus *and 0.2% (n = 4) were *Anopheles implexus*. Due to the small number of *Anopheles arabiensis *(n = 17) identified by PCR, the *An. arabiensis *and *An. gambiae s.s*. were pooled together.

Among the larvae collected, about 98.5% (n = 1012) of *An. gambiae *and all *An. funestus *were collected from the stream areas. A large proportion of *An. gambiae *(52.2%) and *An. funestus *(73.2%) were collected from the drainage ditch habitats. Other habitat types in the stream area where *An. gambiae *was found include man made pools (22.2%), natural swamps (17.1%) and hoof footprints (8.5%). Only fifteen larvae of *An. gambiae *were found in the hilltop area, specifically, in four tire track habitats. No *An. funestus *larvae were collected from hilltops. A total of twenty-seven *An. gambiae *pupae were found in the stream areas.

### Effect of rainfall and topography on habitat stability

In general, the trend in aquatic habitats closely followed the pattern of rainfall; peak habitat frequencies were concurrent with peak rainfall (Figure 2). However, the extent to which habitat frequencies fluctuated between seasons varied greatly between the two topographical regions. While the frequencies of aquatic habitats in the stream area remained relatively static over the study period (fluctuating by no more than 20%), habitat frequency fluctuated by up to 100% in the hilltop areas.

Concordantly, the mean length of days that a habitat remained aquatic was significantly shorter in the hilltop area compared to the stream areas (16.7 d vs 50.2 d during rain and 15.7 d vs 64.0 d after rain) (Table 1). The mean number of times that a habitat dried up in the hilltop area was double that of the stream areas during the rainy season (3.8 vs 2.0), and six times more than in the stream areas after the rainy season (2.6 vs 0.4) (Table 1). Overall, the percentage of habitats that were aquatic on any given day in the hilltop area dropped significantly from 63.77% during the rainy season to 44.62% after the rainy season, whereas these percentages remained unchanged in the stream area regardless of season (91.8% during rain season vs 89.9% after rain season) (Table 1).

**Table 1:** Repeated measure MANOVA comparison of habitat stability between hilltop and stream areas during and after the rainy season.

**Parameters (mean ± SE)**	**During rain**		**After rain**	
		
	**Hilltop**	**Stream area**	**Hilltop**	**Stream area**
Number of days surveyed	121		84	
Number of habitats surveyed	32	55	47	81
Mean duration that habitat remains aquatic (days)	16.71 ± 2.39 A	50.19 ± 2.65 B	15.68 ± 2.45 a	63.98 ± 2.83 b
Mean number of times that habitat dried up	3.75 ± 0.22 A	1.96 ± 0.17 B	2.62 ± 0.18 a	0.42 ± 0.14 b
Mean daily percentage that habitats were aquatic	63.77 ± 3.49 A	91.76 ± 3.49 B	44.62 ± 4.59 C	89.90 ± 4.59 B

Columns not connected by the same letter are significantly different at a level of α = 0.05 (Tukey-Kramer HSD test), lower-case and capital letters indicate independent tests

**Figure 3:**
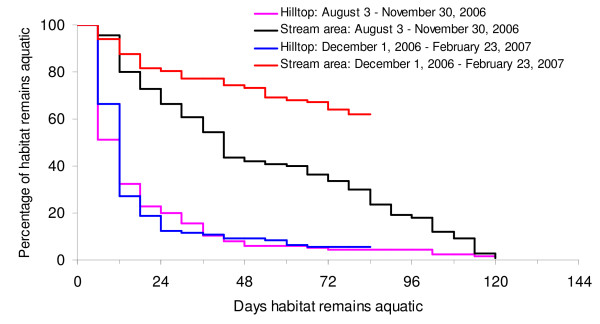
**Kaplan-Meier curve of survivorship of aquatic habitats in different topographic areas and different seasons**. Observations lasted for 120 d during the rainy season and 84 d after the rainy season.

In total, habitats remained aquatic for 12 d or more only about 30% of the time in hilltops. The significantly greater longevity of habitats in the stream areas is evident from Figure 3 (χ^2 ^= 856.12, d.f. = 119, P < 0.001 during the rainy season and χ^2 ^= 1300.98, d.f. = 83, P < 0.001 after the rainy season).

### Effect of topography and rainfall on the occurrences of malaria vectors

Overall, malaria vector larvae and pupae occurred mostly in the stream areas. Larvae of *An. gambiae *were only observed four times in the hilltops (in January 2007). Each time we only found one larvae positive habitat and none of these larvae developed into pupae. Pre-adult stages of *An funestus *were not observed in the hilltop areas throughout the entire study period.

**Figure 4:**
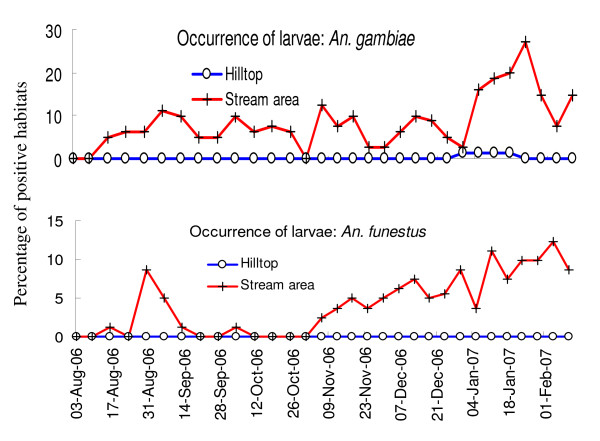
**Temporal dynamics of *Anopheles *larvae of different species in different topographic areas**.

Larvae of *An. gambiae *were consistently found in the stream areas throughout the study period (Figure 4). However, significantly fewer habitats were found to be larval positive during the rainy season than after (5.91% vs. 12.21%, repeated measure MANOVA Tukey-Kramer HSD test, P < 0.05). *Anopheles gambiae *pupae occurrence frequencies were much lower and so no significant difference was found between topographies or seasons (0.85% vs. 0.57% during and after rainy season, Tukey-Kramer HSD test, P > 0.05). The mean density of *An. gambiae *larvae was significantly higher in the stream areas (mean of 0.27 per dip, 95% CI [0.21, 0.32]) compared to the hilltops (mean of 0.02, 95% CI [0.00, 0.10]). The 1.95% prevalence of larval *An. funestus *in the stream areas during the rainy season was shown to significantly increase to 7.66% after the rainy season (Figure 4, Tukey-Kramer HSD test, P < 0.05). The occurrence of *An. funestus *pupae was negligible, with only three sightings within the stream areas in total.

The results of logistic regression analyses indicated that the occurrence (presence/absence) of *An. gambiae *larvae was significantly and positively associated with both the topography (hilltops = 0 and stream area = 1) and the number of days that a habitat remained aquatic, but negatively associated with habitat surface area (χ^2 ^= 84.447. d.f. = 3, *P *< 0.0001). The effect of habitat types and land use types on the occurrence of mosquito larval was not significant.

### Adult mosquito dynamics

**Figure 5:**
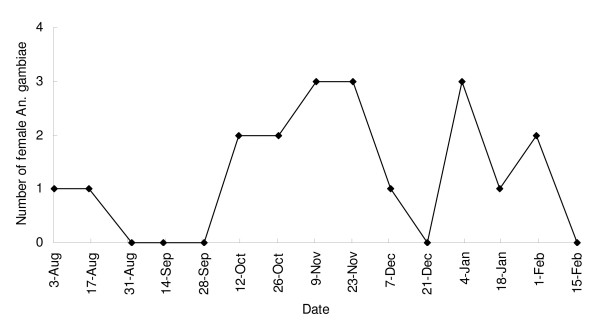
**Dynamics of adult *An. gambiae *by indoor PSC methods**.

During the study period, adult *An. gambiae *appeared sporadically in the area (Figure 5), and were only found inside one or two houses during each survey. A total of 21 female adult *An. gambiae *were collected during the sampling period, and the mean density was estimated at 0.06 females per house per survey. Only one adult *An. funestus *was ever collected (mid-December, 2006).

## Discussion

The present study aimed to investigate the occurrence of malaria vector larvae in relation to topography and habitat stability in order to assess the applicability of a more targeted larval vector control in the highlands of western Kenya. Specifically, the intention was to measure the malaria vector productive potential of breeding sites located in high altitude regions (1,500-1,600 m above sea level). Breeding site productivity studies normally take place at lower altitudes or major river valley area where both pre-adult and adult vectors are found at higher, and more consistent frequencies [2,4,14,16,24]. However, these studies fail to consider critical aspects of vector ecology at the higher altitudes, where human populations are more crowded and more immunologically naïve to infection.

As expected, we found more vector breeding sites nearby streams than along hilltops in our study. The relatively few breeding sites that were found along hilltops greatly fluctuated in frequency between seasons and were highly dependent on rainfall. The increased stability of breeding sites found within the stream areas is simply a result of the more permissive conditions these areas provide for water accumulation [15,25]. Not only was the duration of breeding site viability significantly reduced in the hilltops, but the frequency of anopheline larvae positive habitats was also significantly lower. Very few *An. gambiae *larvae were found in hilltop breeding sites, with a zero pupation rate. Both hilltops and stream areas received equal rainfall, were of identical temperature and had very similar land use. The significant variation between the vector productivity of the two topographically distinct areas is attributed to the significant differences in habitat stability.

Similar observations have been reported in lowlands area of the Lake Victoria Basin in western Kenya, where habitat stability was positively correlated with larval density and pupal productivity [5]. As the Basin is very flat, *Anopheles *larval habitats are widely dispersed and sufficiently stable to be productive even hundreds of meters from the lake [5,24]. The hilly landscape at higher altitudes of the western Kenyan highlands only allows for smaller, discrete areas of sustained water accumulation that are mostly located nearby streams [26-28]. These constitute hotspots for productive anopheline habitats which, given their confined spatiality, might justify localized larval control.

The superior capacity of *An. gambiae *as a colonizer of new territory as illustrated by our study corroborates what is already known for this species [29-32]. Adult malaria vectors were routinely collected from human dwellings of the uphill sites, albeit at low densities. While the uphill habitats appear to be inappropriate targets for vector control, the stream areas of the western Kenyan highlands appear to act as stepping stones between the valleys and the regions of high altitude. There is a tendency for vectors to move to higher altitudes following the rainy season as a result of the increased availability of breeding sites in these areas. Therefore, in eliminating the stepping stone provided by the habitats of the stream areas at high altitude, the extended adult vector distribution might be prevented and the threat of epidemic malaria in the western Kenyan highlands removed.

## Competing interests

The authors declare that they have no competing interests.

## Authors' contributions

YH carried out the field surveys, assembled data and drafted the manuscript. GZ performed statistical analysis and revised the manuscript. LY revised the manuscript. YA, SM and HA participated in the field surveys. AR revised the manuscript. AG and GY participated in the study design and coordination. All authors read and approved the final manuscript.
